# Supplementary figures and images for: High-Depth Transcriptome Reveals Differences in Natural Haploid Ginkgo biloba L. Due to the Effect of Reduced Gene Dosage
Source: Int J Mol Sci. 2022 Aug 11;23(16):8958. doi: 10.3390/ijms23168958 (PMC9409250; doi:10.3390/ijms23168958)

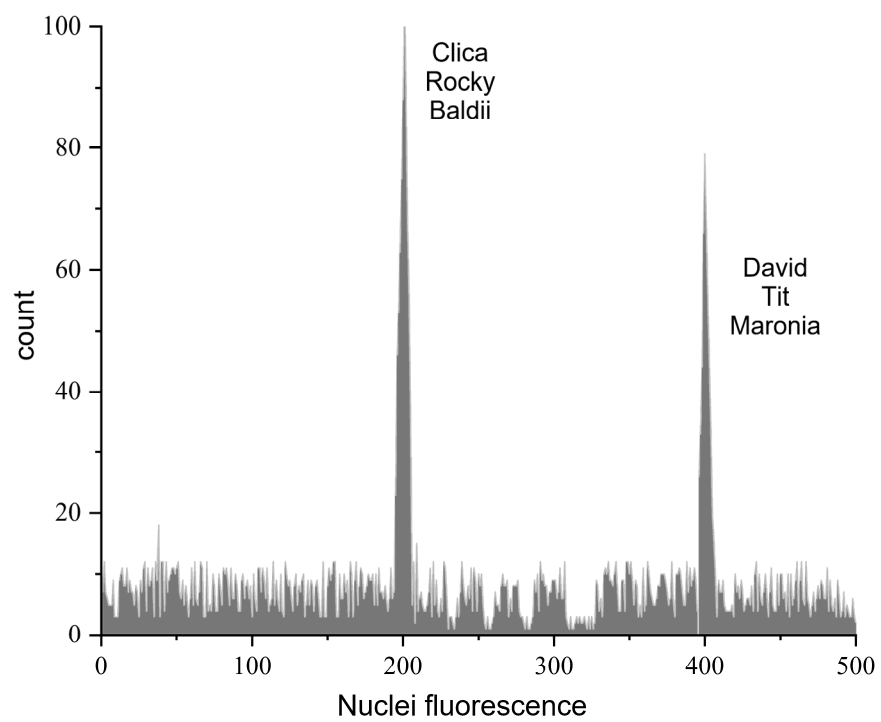

Figure S1. Flow cytometry estimation of DNA ploidy level showing histogram peaks of ginkgo.

Supplement: Supplementary file 1 [file ijms-23-08958-s001.zip › ijms-1777090-supplementary/Supplementary File/Supplementary File S1.The results of the identification of the ploidy of the material..pdf]
